# Financial Exploitation Vulnerability and Social Isolation in Older Adults: Results From a Longitudinal Survey

**DOI:** 10.1093/geroni/igaa057.094

**Published:** 2020-12-16

**Authors:** Gilbert Gimm, Scott Beach

**Affiliations:** 1 George Mason University, Vienna, Virginia, United States; 2 University of Pittsburgh, Pittsburgh, Pennsylvania, United States

## Abstract

OBJECTIVE: This study examined the prevalence and characteristics of socially isolated older adult participants in a survey funded by National Institute of Aging (NIA), and the association of social isolation with financial exploitation (FE) vulnerability. METHODS: Baseline data were collected from 528 participants (age 60+ years) and recruited from two regions. Assessments included a detailed cognitive battery and an array of self-report measures including socio-demographic and psychosocial variables. Social isolation was measured with the Friendship Scale (Hawthorne, 2006). Participants also read six randomly ordered “scam” scenarios (investment opportunity; Medicare phishing and prescription drug fraud; health product; sweepstakes; telemarketing) and provided credibility ratings for each (1 = Not at all credible; 10 = Extremely credible). Ratings are coded “0” (credibility rating 1); “1” (ratings 2-4); “2” (ratings 5-10) and summed, with total FE “vulnerability” scores ranging from 0-12. RESULTS: Preliminary results showed the prevalence of social isolation was 12.5% overall. Social isolation was higher for males (17.4% vs. 9.8%, p<.05); single / never married (28.7% vs. 12.5%, p<.01); and those in the lowest household income (under $10,000) category (28.3% vs. 12.5%, p<.05). However, we did not find evidence that social isolation varied significantly by race or educational attainment. FE vulnerability risk was higher for socially isolated older adults (2.44 vs. 1.44, p<.001) compared to non-isolated participants. CONCLUSION: Social isolation is associated with FE vulnerability. Therefore, identifying the prevalence and characteristics of socially isolated older adults is needed to improve the targeting of interventions for those at greater risk of FE vulnerability.

